# Human-induced pheromone pollution leads to changes in alternative mating tactics of moths

**DOI:** 10.1093/beheco/araf010

**Published:** 2025-02-12

**Authors:** Shevy Waner Rips, Michal Motro, Uzi Motro, Oren Kolodny, Ally Harari

**Affiliations:** Department of Entomology, Volcani Institute, Agricultural Research Organization, Hamaccabim St., Rishon LeZion, 7505101, Israel; Department of Ecology, Evolution and Behavior, The Hebrew University of Jerusalem, Edmond J. Safra Campus, Jerusalem, 9090401, Israel; David Yellin College of Education, Maagal Beit Hamidrash 7, PO Box 3578, Jerusalem, 91035, Israel; Department of Ecology, Evolution and Behavior, The Hebrew University of Jerusalem, Edmond J. Safra Campus, Jerusalem, 9090401, Israel; Federmann Center for the Study of Rationality, The Hebrew University of Jerusalem, Edmond J. Safra Campus, Jerusalem, 9090401, Israel; Department of Ecology, Evolution and Behavior, The Hebrew University of Jerusalem, Edmond J. Safra Campus, Jerusalem, 9090401, Israel; Federmann Center for the Study of Rationality, The Hebrew University of Jerusalem, Edmond J. Safra Campus, Jerusalem, 9090401, Israel; Department of Entomology, Volcani Institute, Agricultural Research Organization, Hamaccabim St., Rishon LeZion, 7505101, Israel; Department of Biology and Environment, University of Haifa at Oranim, Road 7213, Tivon, 3600600, Israel

**Keywords:** alternative mating tactics, chemical pollution, environmental changes, HIREC, mating disruption, pheromone, pink bollworm moth

## Abstract

Environmental changes driven by anthropogenic activities often disrupt animal communication and mating behavior. Consequently, these changes may force animals to adopt alternative mating tactics and strategies to find a mate. The mating disruption technique is an environmentally friendly tactic often used to control the pink-bollworm moth population in cotton fields. Though mating disruption is eco-friendly, it represents a Human-Induced Rapid Environmental Change for the targeted moths. Mating disruption involves spreading a synthetic version of the species-specific sex pheromone in the field, creating a pheromone-polluted environment, making it difficult for male moths to locate females and thereby reducing mating rates. We hypothesized that the intense sexual selection and environmental changes affecting communication would lead moths to increase their use of alternative mating strategies. An observed alternative mating behavior in male pink bollworm moths is disturbing mating pairs to displace the male and mate with the female. We compared this behavior between two populations and found that males long exposed to mating disruption disturbed mating pairs more frequently than those never exposed to it. In addition, males with a prolonged history of exposure to mating disruption showed reduced choosiness of females and increased their mating rate with small females of lower reproductive potential. The success rate of the observed couple disturbance was low. Nonetheless, this strategy, alongside other strategies, may contribute to the males’ reproductive success when facing the additional challenge of locating females due to the pheromone-polluted environment.

## Teaser

Mating disruption is an environmentally friendly pest control technique, used to control insect pests. Focusing on the pink bollworm moth, a key cotton pest, our study examines how males adapt their mating strategies to overcome this challenge. We provide insights into behavioral adaptations to the mating disruption that constitutes a Human Induced Rapid Environmental Change for the moths.

## Introduction

Mating systems refer to the ways in which individuals within a population achieve reproductive success, including the strategies they use to acquire mates. Environmental changes driven by anthropogenic activities often affect the factors influencing variation in mating systems (Lane et al. 2011). These changes include Human-Induced Rapid Environmental Changes (HIREC) such as habitat fragmentation, pollution, and resource reduction. Pollution often diminishes the efficacy of sexual signals (Lürling and Scheffer 2007). For instance, shipping activities produce noise pollution that impairs the ability of Lusitanian toadfish (*Halobatrachus didactylus*) to detect courtship calls (Vasconcelos et al. 2007). Similarly, light pollution disrupts the ability of male glowworms (*Lampyris noctiluca*) to detect female signals (Bird and Parker 2014). Exposure to chemical pollution from organotin compounds disrupts normal reproductive behavior and pheromone communication in snails (*Ilyanassa obsoleta*) (Straw and Rittschof 2004). Consequently, these environmental changes can alter an individual’s fitness payoff, increasing the need to adopt or enhance the use of alternative mating tactics to locate a mate (Isvaran 2005; Mulrey et al. 2015).

Alternative mating tactics aim to maximize fitness in intrasexual competition. Gross (1996) proposed to classify alternative mating tactics into three categories: (1) *Alternative Strategies*, involving genetically fixed phenotypes with equal fitness maintained by frequency-dependent selection; (2) *Mixed Strategies*, where individuals express multiple phenotypes probabilistically; and (3) *Conditional Strategies*, in which individuals flexibly choose tactics based on their condition or environmental context to optimize fitness. Conditional strategies are the most prevalent and enable adaptation to dynamic environments (Gross 1996). Examples of alternative mating behaviors include “sneaker” males that ambush females to avoid direct competition with stronger males (Cade 1980; Ovaska and Hunte 1992; Rowell and Cade 1993; Emlen 1997; Hanlon 1998; Correa et al. 2003; Isvaran 2005; Taborsky et al. 2008; Shuster 2010). Another tactic involves male mimicry of females, as observed in the bluegill sunfish (*Lepomis macrochirus*), where males either defend nests or adopt sneaking and female mimicry tactics (Neff and Svensson 2013). *Panorpa* scorpionflies provide a particularly striking example of conditional strategies, with the tactics employed varying not only by male condition but also by interspecies competition. Thornhill (1987) described how larger males of *Panorpa* present dead arthropod nuptial gifts to attract females, medium-sized males offer saliva masses, and smaller males resort to forced copulation. Usually presenting dead arthropods results in the highest number of matings. In the presence of competition from *Panorpa mirabilis*, which dominates access to crickets used as nuptial gifts, *P. latipennis* males of all sizes increase their reliance on forced copulation, though this tactic remains predominantly used by smaller males. Under these competitive conditions, forced copulation becomes the most effective tactic (Thornhill 1987).

The flexibility of conditional strategies allows individuals to adapt to environmental heterogeneity. For instance, in the damselfly *Protoneura amatoria*, the use of different mating tactics varies with light conditions. Under low light, males tend to wait in the canopy, attempting to mate with unmated females whereas under high light, males more frequently hover over water and harass pairs or females ovipositing on debris (Larison 2007). Human-induced environmental changes can similarly influence mating tactics, as observed in *Salamis parhassus* butterflies, where habitat degradation prompted males to shift from perching to patrolling behavior (Dries and Van Dyck 2009). These examples demonstrate the impact of environmental changes on the success and prevalence of alternative mating tactics.

By influencing the ability of individuals to attract and locate mates, environmental changes may also affect mate choice behavior. Darwin (1871) noted that males typically compete for females, while females are generally choosier due to their higher reproductive investment (Trivers 1972). However, when males invest more in offspring, they may become choosier, as seen in species where males provide costly courtship or nuptial gifts (Gwynne 1981; Rutowski 1982; Edward and Chapman 2011; Fritzsche et al. 2021). Selective males are often observed in insects, including Lepidoptera, where males contribute beyond sperm provision (Bonduriansky 2001). Choosiness may be reduced when its associated costs are high, due to a lower chance of encountering a mate (Jennions and Petrie 1997). For example, female *Teleogryllus oceanicus* crickets reduced choosiness when males were scarce, (Bailey and Zuk 2008) and black widow female spiders (*Latrodectus hesperus*) reduced choosiness when late to encounter a male (Scott et al. 2020).

Mating disruption is an environmentally friendly pest control technique used to manage moths and other insect pests that communicate via pheromones (Cardé 1990; Cardé and Minks 1995; Rizvi et al. 2021). Nevertheless, this eco-friendly method brings about a significant Human-Induced Rapid Environmental Change (HIREC) for the targeted insects. Male moths typically locate females by following the pheromone plume emitted by calling females (Greenfield 1981; Cardé and Haynes 2004; Chen et al. 2021; Golov et al. 2022). The mating disruption technique involves releasing synthetic sex pheromones into target fields, creating an excess of pheromone that disrupts the moths’ chemical communication and impairs the males’ ability to find conspecific females (Cardé 1990). Similar to other rapid environmental changes such as noise and light pollution, mating disruption constitutes a form of chemical pollution that significantly interferes with insects’ mating communication systems. This may lead to the pest population decline, or may drive evolutionary adaptations to overcome the new challenge (Sih et al. 2016).

The pink-bollworm moth, *Pectinophora gossypiella,* is believed to have originated in Asia, but it has since spread to most cotton-producing regions around the world (Naranjo et al. 2002). This moth is one of the most destructive cotton insects (Busck 1917). Many additional insect species attack cotton (Gaines 1957), however, the pink bollworm is especially challenging to control since its larvae feed within flower buds and bolls, where they are protected from conventional insecticides (Cardé and Minks 1995). The mating disruption technique has been used worldwide to control the pink-bollworm moth populations for many years often reducing reliance on conventional insecticides. However in some cases, conventional insecticides targeting eggs and adult moths are applied alongside this technique for additional control (Gaston et al. 1977; Staten et al. 1987; Critchley et al. 1991; Cardé and Minks 1995; Kehat et al. 1999; Lykouressis et al. 2005). Mating disruption is implemented by placing dispensers that release high doses of the species-specific synthetic pheromone, making it difficult for males to locate females in the cotton fields (Staten et al. 1987). While this method has effectively controlled the pink bollworm moth population in cotton fields, recent claims suggest a reduction in its efficiency (Harari and Sharon 2022).

Chemical communication plays an essential role in the pink-bollworm moth’s courtship behavior, like many species of nocturnal moths (Greenfield 1981; McNeil 1991; Groot et al. 2016; Harari and Sharon 2017). The female moth releases a volatile sex pheromone, eliciting premating behaviors from conspecific males. The pheromone of the pink bollworm consists of a 1:1 mixture of Z,Z- and Z,E-isomers of 7,11-hexadecadienyl acetate (Hummel et al. 1973; Bierl et al. 1974). Younger, larger, and well-nourished females exhibit a slight bias in the ratio of the two components, resulting in an increased proportion of the Z,Z component. These females are more fecund and are preferred by males (Gonzalez-Karlsson et al. 2021). Changes in this ratio were recently found in a field population in which the mating disruption technique was applied, suggesting that this may be a means by which moths cope with their new pheromone-polluted environment (Dou et al. 2019). Here, we propose that, similar to situations where environmental changes caused by anthropogenic activity disrupt sexual communication and impair males’ ability to locate females, male pink bollworm moths may adapt to the constraints of the mating disruption technique by altering or intensifying their use of alternative mating strategies to increase their chances of finding females. When searching for calling receptive females, male pink bollworm moths employ an alternative mating tactic where they are also attracted to mating couples (Ouye and Butt 1962). These males disturb the mating couple by flying on and around the couple, trying to separate the mating pair in order to mate with the receptive female (personal observations). We hypothesized that the alternative mating tactic, referred to here as “disturbance behavior,” would increase in moth populations subjected to prolonged exposure to mating disruption, due to the difficulty in locating calling females masked by the excess synthetic pheromone. We also hypothesized that males’ choosiness will decrease in this population due to the “scarcity” of females experienced by mate searching males. In order to test these hypotheses, we measured the frequency of the disturbance behavior for both naïve moths (moths from the population that never experienced the mating disruption technique) and moths from the population exposed to mating disruption for many generations. As an indication of the males’ choosiness, we tested the percentage of males that mated with small, less desirable females amongst the naïve moths and amongst those exposed to mating disruption for many generations. The plasticity of the males’ disturbance behavior and their choosiness was tested by comparing these behaviors in the presence and in the absence of the synthetic pheromone for both populations.

## Methods

### Breeding and maintenance

Two populations of pink bollworm moths were kept in separate climatic rooms at 25 ± 1 °C, 14:10 L:D, 60% RH, in the Department of Entomology, the Volcani Institute, Israel:

(1) A lab population of moths that had never experienced mating disruption (**hereafter, naïve moths**), and (2) moths reared in the lab for 8 to 13 generations that originated from collected cotton bolls infected with the pink bollworm larvae from a field with ±40 yr’ history of applying the mating disruption method (using Shin-Etsu pheromone dispensers). These moths were continuously maintained under a pheromone-saturated environment, using mating disruption pheromone dispensers (**hereafter, prolonged pheromone-exposed moths**). The larvae of both populations were reared on an artificial diet (Stonefly Heliothis Diet, Ward’s Science). Adults were fed on 10% sugar solution ad libitum.

Males and females were assorted into separate cages to prevent mating. The gender of the moths was determined based on the presence/lack of a black line on the sixth abdominal segment in the last larval stage, representing the developing testicles in males. Female pupae were separated and caged apart according to two size groups: large females (0.0170 to 0.0220 g) and small females (0.0070 to 0.0110 g). Medium-sized females were discarded. The adult moths (males, large females, and small females) were further separated daily into age-based cages.

### Experimental design

We caged 10 large females and 10 small females, with an excess of males (35 to 40). All moths used in the experiment were virgins, aged 3 to 5 d, and continuously fed from the day of hatching. Using fluorescent powders that previously has been demonstrated to be harmless and not affecting moth behavior, we marked females and males with a small amount of different colors to distinguish between the sexes during the experiment. Each trial took place 3 to 4 h after the onset of the dark period, for a duration of 1 h. In order to quantify the occurrence of the male disturbance behavior, we (1) recorded the number of mating couples, every 5 min, and summed up these numbers to obtain the *mating intensity*, and (2) continuously recorded the number of males disturbing any of the mating couples to obtain the *number of disturbances*. In order to estimate male *choosiness*, the size of the mated female was recorded for each couple.

### Treatments description

We conducted the experiment described above for the following four groups of moths:

(1) Moths from a naïve population, placed with pheromone dispensers during the experiment (*n* = 9 trials).
(2) Moths from a naïve population (which had never been exposed to mating disruption), studied without exposure to synthetic pheromones dispensers during the experiment (*n *= 10 trials).
(3) Prolonged pheromone-exposed moths, placed with pheromone dispensers during the experiment (*n* = 10 trials).
(4) Prolonged pheromone-exposed moths, not exposed to synthetic pheromones dispensers during the experiment (*n* = 11 trials).

### Description of the variables measured

Since the males’ disturbance behavior is affected by the number of mating couples, we tested the disturbance behavior in relation to the mating intensity:

Mating intensity: The number of mating couples recorded every 5 min, summed over the entire trial.

Number of male disturbances: The number of instances where a male arrived at a mating couple and attempted to separate them, either by flying around or directly on the couple. This male disturbance behavior was noted regardless of the duration the male engaged in the disturbance until he left the couple.

Female size: Categorization of each mating female as either large or small.

We compared (1) mating intensity, (2) the frequency of male disturbance events relative to mating intensity, and (3) the degree of male mating choosiness regarding female size, each was compared across the four treatment groups.

### Statistical analysis

Two-way ANOVA tests were applied to test whether either or both the origin and the presence of synthetic pheromone influenced mating intensity and male disturbance behavior. Since neither of these two variables have a normal distribution, we applied the square-root transformation on each and obtained normality.

We considered a disturbance event as successful if the disturbing male succeeded to disrupt the mating couple and to separate the male from the female. To calculate the Rate of Success, we divided the total number of successful disturbances by the total number of disturbances (each total is summed over all relevant trials of a treatment). Note that this is actually a ratio between two random variables (the number of successful disturbances and the total number of disturbances). Thus, this ratio does not follow a simple binomial distribution. For the analysis of this ratio we used the ratio estimate technique. This solves the problem of some trials having a zero number of disturbances. Since our variables do not have a normal distribution, asymptotic estimates of the ratio’s standard error are not reliable, so these were estimated by bootstrap analyses (eg Choquet et al. 1999). Thus, we performed 10^4^ bootstrap samples, and calculated the ratio for each. The standard deviation of these 10^4^ simulated ratios served as the standard error of the ratio estimate. We applied this method to compare the frequency of successful disturbances between the two moth origins (naïve and prolonged pheromone-exposed) and between the two treatment conditions (with or without exposure to synthetic pheromone dispensers). Statistical Analyses were done with SPSS (IBM Corp. 2023).

To assess differences in male choosiness, we compared the percentage of matings with small females (out of the total number of mating) between naïve moths and the prolonged pheromone-exposed moths, both with and without synthetic pheromone during the experiment. These variables do not have a normal distribution (not even after subjecting them to the commonly used transformations), thus we used the Mann-Whitney non-parametric test for the comparisons.

## Results

### Mating intensity

Mating intensities were as follows: (1) naïve moths exposed to mating disruption (pheromone dispensers) for the first time during the experiment (11.00 ± 1.56) (mean ± se), (2) naïve moths never exposed to mating disruption (18.20 ± 3.06), (3) prolonged pheromone-exposed moths with pheromone dispensers during the experiment (43.00 ± 6.90), and (4) prolonged pheromone-exposed moths without pheromone dispensers during the experiment (64.45 ± 6.59).

The mating intensity was significantly influenced by both the origin (naïve or prolonged pheromone-exposed moths) and by the presence or absence of the synthetic pheromone during the experiment (Two-way ANOVA; Origin: *F*_1,36_ = 73.069, *p* < 0.001, Pheromone: *F*_1,36_ = 8.967, *p* = 0.005, Interaction: *F*_1,36_ = 0.671, *p* = 0.418).

**Fig. 1.**
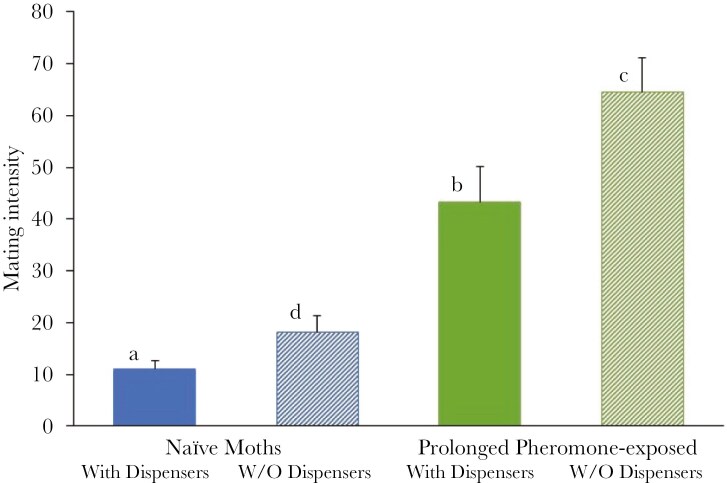
Mating intensity in each of the four treatments. The columns represent the mean (+ se) per trial. Different letters denote significant differences at the 5% level (considering the Bonferroni correction for multiple comparisons).

A greater number of prolonged pheromone-exposed moths mated compared to the naïve moths. The presence of the synthetic pheromone (ie mating disruption) during the experiment had a significant influence on the mating intensity of moths that have been exposed to mating disruption for many generations. These moths mated more when pheromone dispensers were absent, and the additional challenge of locating mates was reduced. However, for the naïve moths, the encounter with appliance of pheromone dispensers for the first time within the given hour of the experiment did not significantly reduce the number of mating couples (Fig. 1).

### Male disturbance behavior

Male disturbance events in relation to the mating intensity were as follows: (1) naïve moths exposed to mating disruption (pheromone dispensers) for the first time during the experiment (11.45% ± 4.15%) (mean ± se), (2) naïve moths never exposed to mating disruption (28.85% ± 8.20%), (3) prolonged pheromone-exposed moths with pheromone dispensers also during the experiment (78.14% ± 13.90%), and (4) prolonged pheromone-exposed moths without pheromone dispensers during the experiment (47.18% ± 6.00%).

The male disturbance behavior was influenced by the origin (naïve or prolonged pheromone-exposed moths). The disturbance behavior was not affected by the presence or absence of the synthetic pheromone during the experiment per se, although a strong interaction between the origin and the presence of pheromone dispensers was revealed (Two-way ANOVA; Origin: *F*_1,36_ = 31.509, *p* < 0.001, Pheromone: *F*_1,36_ = 0.115, *p* = 0.736, Interaction: *F*_1,36_ = 8.046, *p* = 0.007)

**Fig. 2.**
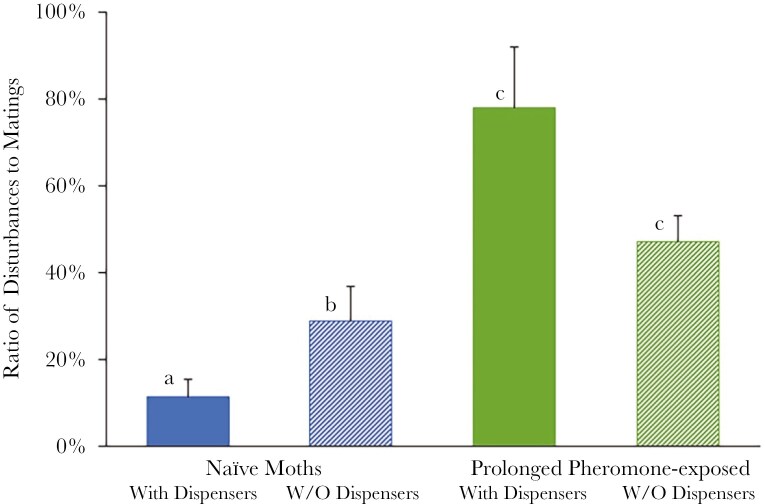
Percentage of matings experiencing disturbance by other males. The columns represent the mean (+se) per trial. Different letters denote significant differences at the 5% level (considering the Bonferroni correction for multiple comparisons).

Males exposed to mating disruption for many generations disturbed mating couples significantly more than naïve moths. When pheromone dispensers were added during the experiment, there was a strong significant negative interaction between the tendency of the disturbance behavior of the two populations; In the presence of the synthetic pheromone during the experiment, there was an increase (although not significant) in the disturbance behavior practiced by the prolonged pheromone-exposed moths, while the disturbance behavior was significantly reduced in the naïve moths (Fig. 2).

The success rate of the disturbance behavior, in which a male successfully disrupted a mating couple and separated the mating male from the female, is presented in Table 1.

**Table 1.** Mating intensity, disturbance events and successful disturbances in each of the four treatments. The first row of each cell presents the mating intensity. The second row presents the number of disturbance events and in parentheses this number divided by the mating intensity. The third row presents the number of successful disturbances and in parentheses the percent of disturbances which turned to be successful.

		With Pheromone		Without Pheromone		Total	
Naïve	Mating Intensity Disturbances Successes	99 13 0	(0.131) (0.00%)	182 52 2	(0.286) (3.85%)	281 65 2	(0.231) (3.08%)
Prolonged Pheromone-exposed	Mating Intensity Disturbances Successes	432 312 16	(0.722) (5.13%)	709 334 23	(0.471) (6.89%)	1141 646 39	(0.566) (6.04%)
Total	Mating Intensity Disturbances Successes	531 325 16	(0.612) (4.92%)	891 386 25	(0.433) (6.48%)	1422 711 41	(0.500) (5.77%)

The overall success rate was 5.77% ± 0.99% (mean ± se). The success rate of naïve males (3.08%) was not significantly different from that of males from prolonged pheromone-exposed population (6.04%) (estimated *p* = 0.142 by the bootstrap analysis). The success rate did not differ between the two treatment conditions, with (4.92%) or without exposure to the pheromones dispensers (6.48%) (estimated *p* = 0.426 by the bootstrap analysis).

### Male choosiness

The males’ choosiness, based on the females’ size, was significantly affected by the moths’ origin. The percentage of small mated females (from all the mated females) was 9.90% ± 4.26% (mean ± se) among naïve moths and 21.92% ± 3.84% among moths exposed to pheromone dispensers for many generations (*p* = 0.002, Mann-Whitney *U* test). There was no significant difference between the percentage of small mated females when pheromone dispensers were present (16.16% ± 5.51%) or absent (16.25% ± 2.87%) during the experiment (*p* = 0.276, Mann-Whitney *U* test).

## Discussion

Environmental changes can affect the ability of individuals to attract and locate mates. To counteract a negative influence on their reproduction, individuals may alter their mating behavior (Van Baaren and Candolin 2018) and change or increase the use of alternative mating tactic (Sinervo and Lively 1996; Shuster 2010). The mating disruption technique, ie the use of high doses of the synthetic pheromones to mask the natural pheromones of calling females, constrains targeted male pests from locating mates and mating (Cardé and Minks 1995). This Human-Induced Rapid Environmental Change can reform the fitness payoff and the use of alternative mating tactics in the population (Isvaran 2005). Males of the pink-bollworm moth are attracted to mating couples in addition to calling females (Ouye and Butt 1962). Taking a female from another male, who has successfully located her despite the pheromone pollution, could allow males to mate with a receptive female otherwise concealed from them. We found that male pink-bollworm moths that originated from the population that had been long exposed to mating disruption disturbed mating couples more frequently than the males from the naïve population that had not been exposed to the mating disruption technique. Interestingly, we found a strong negative interaction between the two origins in the presence of the synthetic pheromone during the experiment. Prolonged pheromone-exposed moths further increased their disturbance mating behavior in the presence of the synthetic pheromone in contrast to naïve moths, which significantly reduced disturbing mating couples when synthetic pheromone was present at the time of the experiment. We suggest that moths exposed to mating disruption for many generations have evolved to increase the use of their disturbance behavior as an alternative mating strategy to overcome the strong reproductive constrain imposed on them. In the absence of the disruptive pheromones, the challenge of locating a mate was abated and males reduced applying this tactic. In contrast, our results suggest that naïve moths who were inexperienced and unfamiliar with this obstacle could not readily adapt by switching to this alternative strategy. Instead, we observed that naïve males reduced their activity in response to the sudden exposure to the synthetic pheromone, including a reduction in the rate of disturbing mating couples. The males’ reduced activity may have occurred due to a reduced sensitivity to the high, constant exposure (sensory adaptation) or due to habituation at a central processing level (Cardé and Minks 1995). This was not apparent when looking at the total number of mating events of naïve males, most likely because the experimental conditions allowed for random mating encounters.

The success rate of disturbing males in separating a mating couple was low. High competition among multiple disturbing males for a mating couple was frequently observed. In such cases, only one male may secure the receptive female, while the others fail. The competition for receptive females in the relatively high-density experimental cages is likely stronger than in the field, making it difficult to accurately assess the mating success of the disturbing males. Therefore, we measured only the success rate of separating the mating couple. The “prior residence advantage,” in which the owner of a resource often succeeds better in competition over that resource (Barnard and Brown 1982; Simmons 1986; Motro et al. 2017) may contribute to the low mating success of a rival male. Even though the disturbance behavior of males brings about only a few rewarding results, this alternative strategy may be worth the effort when locating females becomes increasingly challenging, as is the case in the pheromone-polluted environment.

Alternative mating tactics may be influenced by body size, with smaller individuals potentially adopting these tactics more frequently to secure a mate (Crespi 1988; Leary et al. 2005; Pitnick et al. 2009; Shuster 2010). In this study, we did not explicitly test whether smaller males are more likely to engage in alternative mating strategies compared to larger males; however, observations suggest this not to be the case. Further investigation of this question in future experiments could yield valuable insights and represent a meaningful extension of our current research.

In addition, we hypothesized that males long exposed to mating disruption should reduce their choosiness due to the difficulties in finding females. When environmental changes reduce the availability of mates, animals that maintain their choosiness are expected to suffer a decline in their reproductive success (Møller and Legendre 2001). Therefore, animals are expected to minimize choosiness when they sense a reduction in mating opportunities (Scott et al. 2020). Indeed, we found that males from populations that had experienced long exposure to mating disruption increased their mating rate with smaller, less desired females.

The total number of mating couples was higher for moths from a population continuously exposed to mating disruption than the total number of mating couples amongst the naïve moths. Moths long exposed to mating disruption reduced their choosiness and mated at a higher rate, while naïve moths, never exposed to the strong selection pressure imposed by the mating disruption methods, were choosier and mated at a slower rate. For the prolonged pheromone-exposed moths, the number of mating couples increased when pheromone dispensers were absent during the experiment. This suggests that mating disruption still affects the moths, as corroborated by other studies (Critchley et al. 1983; Cardé et al. 1998; Lykouressis et al. 2005). This result was not obtained, however, for the naïve moths; adding pheromone dispensers during the experiment did not significantly affect the number of mating couples. We suggest that the naïve moths, which were choosier and mated at a slower rate, continued to detect the few female moths they encountered randomly within the confined experimental cage.

The two moth populations we examined were firstly, a group of naïve moths reared in the lab for around 40 yr, which have never been exposed to mating disruption, and secondly, prolonged pheromone-exposed moths originated from fields treated with mating disruption. The latter group was then reared in the lab for 8 to 13 generations before being used in our experiments. Both groups were reared under identical conditions except for the presence of pheromone dispensers (mating disruption) during rearing of the latter. While we made efforts to minimize extraneous factors influencing the experiments there may still be some unknown background noise that could have contributed to the obtained results.

To our knowledge, this is the first research testing the alternative mating behavioral responses of insects to the mating disruption technique. Understanding these behavioral adaptions may also assist in understanding and improving the appliance of this environmentally friendly pest management technique in agricultural ecosystems. Whether alternative behaviors in the pink bollworm moths are sufficient to maintain a population despite intentional HIREC is difficult to determine. Yet, the observed changes in the alternative mating strategies in the pink bollworm may be adaptive on a long-term scale.

Here we present two adaptive behavioral strategies employed by the pink bollworm moths to maximize their mating opportunities, despite the obstacle imposed on them by a pheromone-polluted environment. These strategies involved a reduced selectivity in mating partners and an increased frequency of the disturbance behavior. The success rate of the disturbance behavior of males was low. However together with the reduced choosiness and the observed changes in the ratio of the females’ pheromone components in the pheromone polluted fields (Dou et al. 2019; Gonzalez-Karlsson et al. 2021), these adaptations may allow the males to successfully locate mates despite the mating disruption.

Mating disruption has been utilized across a wide range of insect species for numerous years. As far as we are aware, behavioral adaptations have not been explored in other insect species, presenting a potential area for future investigation.

## Data Availability

Analyses reported in this article can be reproduced using the data provided by Waner Rips et al. (2025).
